# Effects of Water and Nitrogen Addition on Ecosystem Carbon Exchange in a Meadow Steppe

**DOI:** 10.1371/journal.pone.0127695

**Published:** 2015-05-26

**Authors:** Yunbo Wang, Qi Jiang, Zhiming Yang, Wei Sun, Deli Wang

**Affiliations:** 1 Key Laboratory for Vegetation Ecology, Ministry of Education, Institute of Grassland Science, Northeast Normal University, Changchun, Jilin Province, P. R. China; 2 Veterinary and Animal Science College of Heilongjiang Bayi Agricultural University, Daqing, Heilongjiang Province, P. R. China; Chinese Academy of Sciences, CHINA

## Abstract

A changing precipitation regime and increasing nitrogen deposition are likely to have profound impacts on arid and semiarid ecosystem C cycling, which is often constrained by the timing and availability of water and nitrogen. However, little is known about the effects of altered precipitation and nitrogen addition on grassland ecosystem C exchange. We conducted a 3-year field experiment to assess the responses of vegetation composition, ecosystem productivity, and ecosystem C exchange to manipulative water and nitrogen addition in a meadow steppe. Nitrogen addition significantly stimulated aboveground biomass and net ecosystem CO_2_ exchange (NEE), which suggests that nitrogen availability is a primary limiting factor for ecosystem C cycling in the meadow steppe. Water addition had no significant impacts on either ecosystem C exchange or plant biomass, but ecosystem C fluxes showed a strong correlation with early growing season precipitation, rather than whole growing season precipitation, across the 3 experimental years. After we incorporated water addition into the calculation of precipitation regimes, we found that monthly average ecosystem C fluxes correlated more strongly with precipitation frequency than with precipitation amount. These results highlight the importance of precipitation distribution in regulating ecosystem C cycling. Overall, ecosystem C fluxes in the studied ecosystem are highly sensitive to nitrogen deposition, but less sensitive to increased precipitation.

## Introduction

Ecosystem C cycling is an important ecological process in grasslands, which are often constrained by the availability of key resources, such as water and nitrogen [1, 2]. According to the IPCC report [3], alterations in global precipitation regimes are likely to happen in mid-latitude regions, with increased annual precipitation and more frequent extreme rainfall events. Simultaneously, worldwide nitrogen deposition, which has been studied extensively, is likely to increase due to the accelerating industrialization and use of nitrogen fertilizer [4–6]. The predicted alteration in the precipitation regime and enhancement in nitrogen deposition may have a profound influence on grassland ecosystem C fluxes [7], which are highly relevant for future projections of C sequestration in terrestrial ecosystems.

Components of ecosystem C exchange are very sensitive to altered precipitation regimes, including both changes in the amount of precipitation and temporal distribution [8–13]; however, the overall effects of altered precipitation on net ecosystem CO_2_ exchange (NEE) remain highly controversial. In temperate semiarid steppe, water addition enhances NEE, which has been attributed to gross ecosystem productivity (GEP) being more sensitive to altered water availability than ecosystem respiration (ER) [9, 10]. Results of other studies have shown no or negative responses of NEE to water addition [13–15]. The inconsistencies among previous studies may have resulted from differences in climate type and soil texture. The uncertainties in the response are likely to be further complicated by the characteristics of dominant species, which differ substantially in water use efficiency and adaptation to changing soil moisture [16, 17].

In addition to changes in the amount of precipitation, the effects of alteration in the seasonal distribution of precipitation cannot be ignored in grassland ecosystems [16, 18, 19]. Climate change may lead to a dramatic redistribution of precipitation and heat both within and across seasons, causing wide fluctuations in soil moisture [3, 20]. Generally, precipitation will be more efficiently used when its occurrence matches the growth requirements of dominant species [21]. Undoubtedly, seasonal variation in precipitation will increase the risk of uncoupling between water availability and plant community water requirements, and ultimately influence ecosystem C fluxes through reduced plant water use efficiency [19]. Niu et al. (2005) found that the seasonal distribution of precipitation altered photosynthetic characteristics and interspecific competition between C_3_ and C_4_ plants [22]. However, the effects of the altered timing of precipitation distribution on ecosystem C fluxes remain largely unknown. In a drought manipulation experiment, summer drought had more deleterious effects than either spring or autumn drought, which were attributed to stronger competition for limiting resources between plants and microorganisms [23]. Therefore, precipitation distribution needs to be taken into consideration for the interpretation of seasonal and interannual variation in ecosystem C fluxes.

Besides changes in the amount and seasonal distribution of precipitation, variation in rainfall frequency and intensity may also have profound impacts on plant growth and ecosystem carbon exchange by affecting soil infiltration, soil evaporation, etc. [11, 24, 25]. Knapp et al. (2002) found that, without changing the amount of total precipitation, larger intervals between precipitation events reduced ANPP by about 10% in a mesic grassland [26]. Other than its direct influence on ANPP, rainfall intensity can also alter the responses of grassland primary productivity to drought [27]. With the intensification of global climate change, more frequent extreme events will likely to lead to larger but less frequent precipitation events, which may potentially influence ecosystem carbon cycling [28]. More detailed studies are required to understand the effects of altered precipitation frequency and intensity on grassland ecosystem CO_2_ fluxes.

As one of the major limiting factors to plant growth and net primary productivity of terrestrial ecosystems [29], nitrogen addition has often been observed to enhance aboveground productivity and GEP by stimulating plant photosynthesis, increasing the leaf area index (LAI), and improving ecosystem water use efficiency [30–32]. To date, there is no generalized conclusion on the response of NEE to nitrogen addition [33]. For example, Niu et al. (2010) reported a significant positive effect of nitrogen addition on NEE during the first experimental year in a temperate steppe; however, the stimulating effects of nitrogen addition decreased and even diminished in the subsequent experimental years [34]. No effects, or even negative effects, of nitrogen addition have also been reported previously [35, 36]. Long-term fertilization associated with soil acidification and ammonium toxicity are likely the primary causes of these findings [34, 37]. In addition, the effects of nitrogen addition on ecosystem CO_2_ fluxes may also be influenced by vegetation composition, community structure, or other limiting factors, such as precipitation [7, 38].

To understand the impacts of projected changes in both precipitation regimes and nitrogen deposition on the C dynamics of grassland ecosystems, we conducted a water and nitrogen addition experiment in a meadow steppe in northeast China. We measured vegetation composition, biomass, and ecosystem C fluxes for 3 consecutive years that differed greatly in the amount of growing season precipitation. The questions we asked for this study are: 1) how do ecosystem C fluxes respond to water and nitrogen amendments over the experimental years with fluctuating precipitation; 2) what are the impacts of changes in the timing and amount of precipitation on ecosystem CO_2_ exchange; and 3) how important are the precipitation regimes in controlling ecosystem C fluxes in a meadow grassland?

## Materials and Methods

### Ethics Statement

No specific permissions were required for the described field studies, because the Songnen Grassland Ecological Research Station at the Changling Horse Breeding Farm is a department of Northeast Normal University. No specific permissions were required for this study, because it follows the guidelines set by Northeast Normal University. No specific permissions were required for these locations/activities. No location was privately-owned or protected in any way, and the field studies did not involve endangered or protected species.

### Study site

The experiment was conducted in Changling Horse Breeding Farm (44°30′–44°45′N, 123°31′–123°56′E), which is located in Western Jilin Province, northeast China. The studied area has a typical meso-thermal monsoon climate with a mean annual temperature of 6.4°C and mean annual precipitation of 471 mm (1950–2004). The main soil type is classified as chernozem soil with 2.0% soil organic carbon content and 0.15% soil total nitrogen. Vegetation is dominated by *Leymus chinensis*, a C_3_ perennial rhizomatous grass; *Phragmites australis*, *Chloris virgata*, and *Kalimeris integrifolia* are also abundant. However, the C_3_ grasses represent approximately 90% of the total plant biomass [39, 40].

### Experimental design

In the studied grassland, we fenced an area of 100 m × 100 m in 2010. Grazing was excluded from the fenced area. Within the fenced area, 6 blocks (each 20 m × 20 m) were randomly selected. Each block was divided into 4 plots (each 10 m × 10 m) and randomly assigned treatments of control (CK), water addition (W), nitrogen addition (N), and water addition plus nitrogen addition (WN). For the water addition treatment, we added 120 mm water (about 25% of annual mean precipitation) throughout the growing season from 2012 to 2014. The water addition was applied manually as 15 mm of precipitation biweekly from May to September. For the nitrogen addition treatment, 10 g N m^-2^ yr^-1^ (5 g N m^-2^ in both early May and early July) in the form of urea was applied from 2011 to 2014.

### Measurements of climate data

Climate data, including precipitation and air temperature, in 2012, 2013, and 2014 were obtained from an eddy tower roughly 15 km away from the experimental site. Precipitation regime data (monthly precipitation amount, intensity, frequency, and evenness) used in this study were calculated from daily climate data over the growing seasons (from April to October). Monthly precipitation frequency was estimated as the number of days with precipitation greater than 0.5 mm. The monthly mean precipitation interval was calculated as the average number of days between 2 consecutive rainfall events within a month. The monthly precipitation evenness was estimated as the Shannon Index of precipitation [41]:

$$D\;=\;-\frac{\sum_{i\;=\;1}^{n}P_{i}\ln P_{i}}{\ln n}$$

where *D* is the monthly precipitation evenness, *P*_*i*_ is the proportion of daily precipitation in the corresponding monthly precipitation for the i^th^ day, and *n* is the number of days in the corresponding month (*n* = 30 or 31). In this calculation, a tiny amount of precipitation (0.001 mm) was assigned to days without precipitation to meet the requirements for calculation (*P*_*i*_ ≠ 0).

### Vegetation sampling

A vegetation survey was conducted once a month during the growing season from 2012 to 2014. In each plot, vegetation sampling was carried out in 5 randomly placed quadrats (0.5 m × 0.5 m). For each quadrat, we recorded the number of plant species and individuals and the percent cover of each species. The significance of a species in the plant community was assessed by the importance value index (IV), which was calculated as: IV_i_ = (RD_i_ + RF_i_ + RC_i_)/3, where RD_i_, RF_i_, and RC_i_ are the relative density, relative frequency, and relative coverage of species i, respectively.

Aboveground biomass and belowground biomass were measured in late July from 2012 to 2014. For the measurements of aboveground biomass, we harvested plants by clipping at ground level in a 0.5 m × 0.5 m quadrat, which was randomly placed in each plot. Belowground biomass was measured by washing the roots out of a soil core with a diameter of 10 cm and depth of 10 cm. Plants and roots were oven-dried at 70°C to a constant weight.

### Ecosystem carbon flux measurement

Before the start of the experiment (May 2012), an iron frame (0.5 m × 0.5 m) was inserted into the soil to a depth of 5 cm in each subplot. Care was taken to minimize soil disturbance during installation. The iron frames were used to mount a mobile canopy chamber. Ecosystem C fluxes were measured once a month over the growing season (from May to September) in 2012, 2013, and 2014. Under cloud-free conditions, measurements were performed with an infrared gas analyzer (LI-6400, LiCor Inc., Lincoln, NE, USA) attached to a transparent chamber (0.5 m × 0.5 m × 1 m polymethyl methacrylate) from 7 A. M. to 11 A. M. To minimize the effects of short-term changes in environmental conditions on ecosystem C exchange, we measured the 6 blocks sequentially. Within the chamber, 4 small electric fans ran continuously to promote air mixing during the measurement. For each measurement, 11 consecutive recordings of CO_2_ concentration were taken at 10 s intervals during a 2 min period, and these data were subsequently used to calculate net ecosystem CO_2_ exchange (NEE). After the measurement of NEE, the chamber was vented; CO_2_ concentration was measured again using the chamber covered by an opaque cloth in order to calculate ecosystem respiration (ER). Details about the measurements of NEE and ER have been described in a previous study [32]. Gross ecosystem production (GEP) was calculated as: NEE + ER.

### Soil moisture, temperature, and pH

For each growing season, monthly soil moisture and soil temperature at 10 cm depth were determined concurrently with the measurements of ecosystem CO_2_ fluxes. Soil water content was measured by an ECH_2_O soil moisture sensor (EC-5, Decagon Ltd, Pullman, WA, USA) and data were read with a ProCheck (Decagon Ltd, Pullman, WA, USA). Soil temperature was measured by a temperature probe (6000-09TC) coupled to an infrared gas analyzer (LI-6400, LiCor Inc., Lincoln, NE, USA). At the end of July, we collected 3 soil samples from each plot and mixed them together. The mixed soil sample was sieved through a 2-mm mesh and air dried to a constant weight. The soils were suspended in DI water (soil:water = 1:5) and the pH value was determined by a pH meter (PHS-3E INESA Scientific Instrument Co., Ltd, Shanghai, P. R. China).

### Statistical analysis

The reported seasonal mean values were calculated from monthly mean values. Three-way ANOVA analyses were employed to analyze the effects of experimental year, water addition, and nitrogen addition and their interactions on ecosystem C fluxes (NEE, ER, and GEP) and aboveground biomass. As the effect of year was not significant (*P* > 0.05), two-way ANOVAs were performed to assess the main effects of nitrogen and water additions and their interactions on the seasonal mean values of ecosystem C fluxes for each experimental year. We applied a one-way ANOVA and post-hoc test to compare differences among the CK, W, N, and WN plots in NEE, ER, and GEP over the 3 experimental years. Linear regression analysis was used to evaluate the dependence of NEE, ER, and GEP on ANPP or the amount of precipitation in the early growing season. Sigmoid curve estimations were used to evaluate relationships between precipitation regimes (precipitation frequency, precipitation input, individual precipitation input, or precipitation evenness) and ecosystem C fluxes. All statistical analyses were performed using SAS software (SAS 8.0, SAS Institute Inc., Cary, NC, USA).

## Results

### Meteorological data

**Fig 1:**
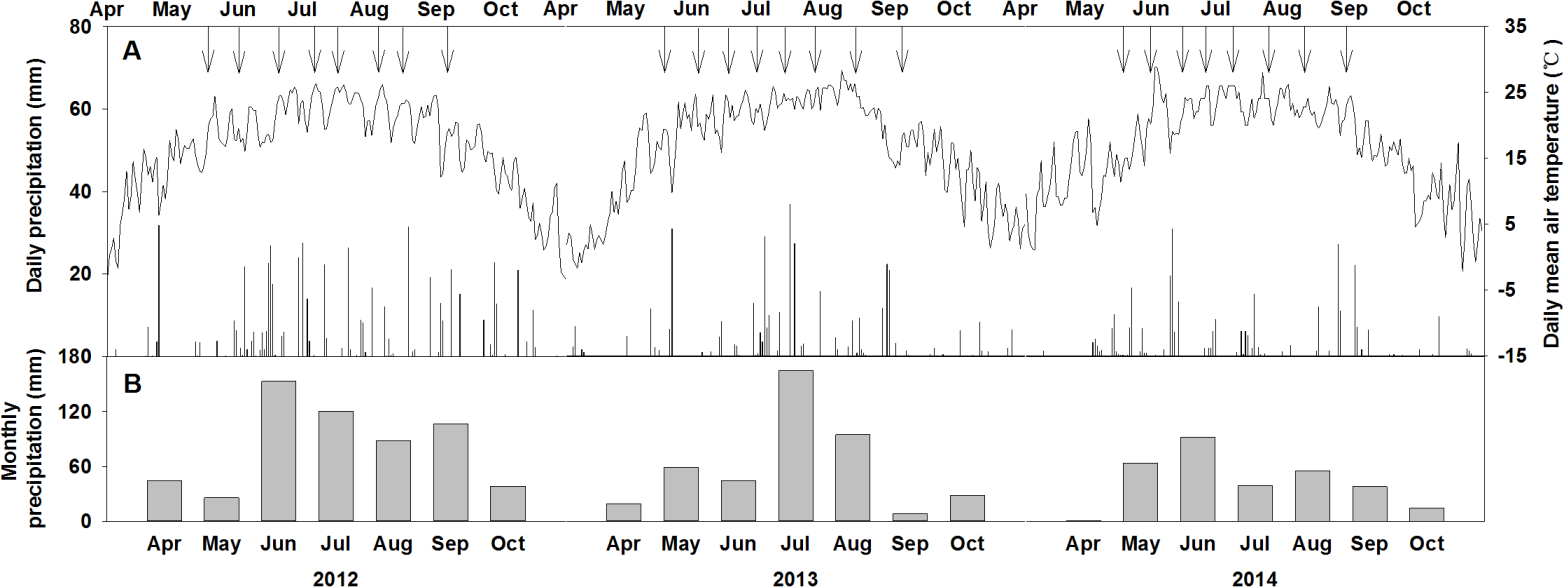
Seasonal patterns of (A) daily precipitation (bars, mm), daily mean air temperature (lines, °C), and (B) monthly precipitation (mm) in 2012, 2013, and 2014. Arrows on top of panel A indicate dates when water addition treatments were applied.

For the 3 experimental years, both the total amount and seasonal distribution of growing season precipitation varied substantially (Fig 1). Growing season precipitation (from April to October) in 2012 (577 mm), 2013 (373 mm), and 2014 (305 mm) was 140%, 90%, and 74% of the long-term average precipitation (411 mm), respectively (Fig 1). There were apparent temporal dynamics in the daily mean air temperature during the growing season with higher temperatures in the summer and lower temperatures in both spring and autumn (Fig 1). For the 3 experimental years, rainy days (daily precipitation > 0.5 mm) from May to September ranged from 38 to 48 and average precipitation intensity varied between 6.8 and 9.8 mm (Fig 1).

### Soil pH, moisture, and temperature

**Fig 2:**
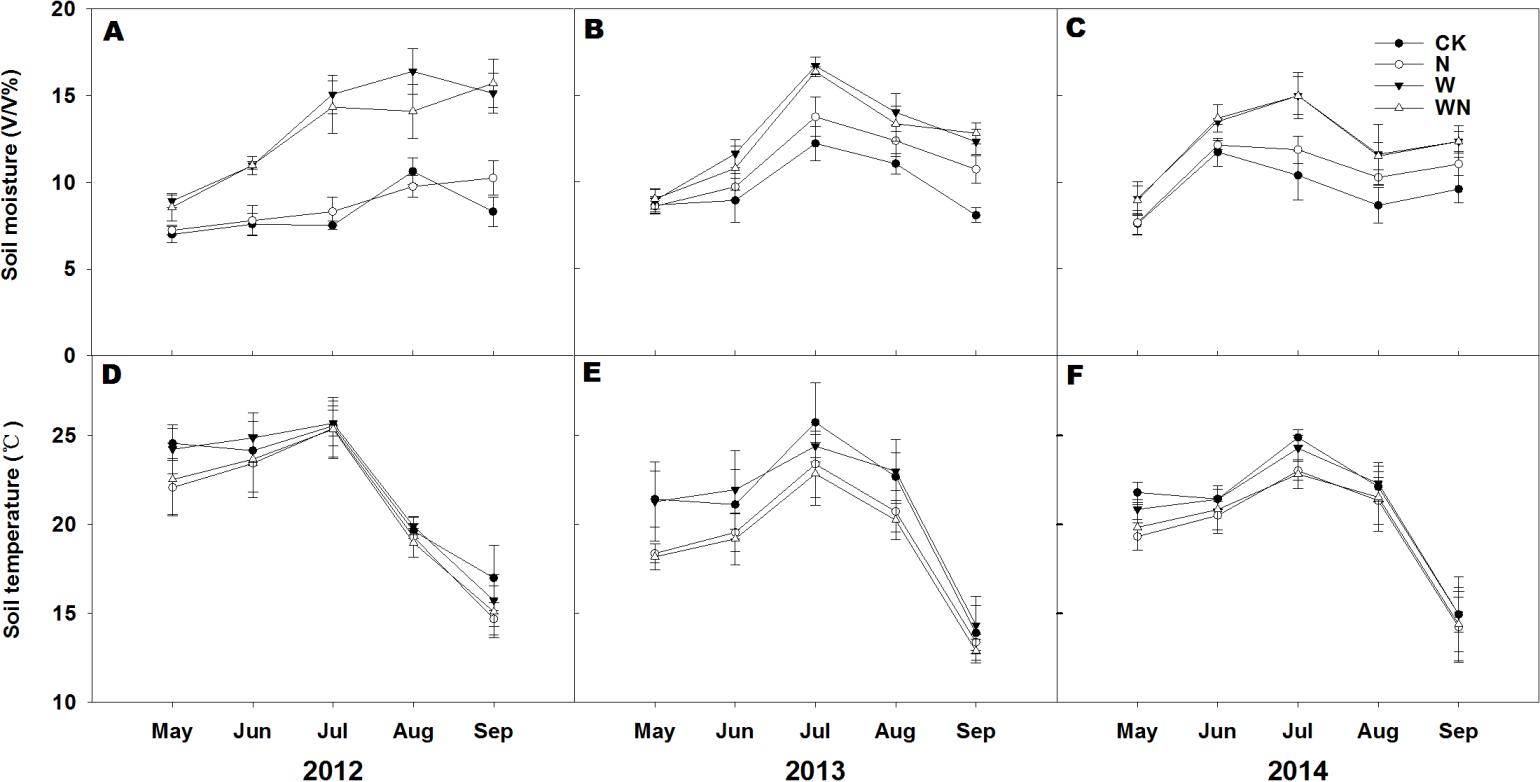
Seasonal dynamics of (A, B, and C) soil moisture (V/V%) and (D, E, and F) soil temperature (°C) at 0–10 cm depth in 2012, 2013, and 2014. CK: control; W: water addition; N: nitrogen addition; WN: water and nitrogen added in combination. Data are reported as mean ± 1 SD (*n* = 6).

Compared to the control (CK) plots, the nitrogen addition (N) and water addition (W) treatments showed no effects on soil pH in 2013 and 2014 (Table 1). For the 3 experimental years, soil moisture in the W and water plus nitrogen addition (WN) plots was greater than in the CK and N plots. There were no differences in soil moisture between either CK and N plots or W and WN plots (Fig 2). The soil temperature at 10 cm depth fluctuated greatly over the growing seasons, with the highest values in July for all experimental years (Fig 2).

**Table 1:** Seasonal means of soil pH (unitless value), vegetation density of the community (VD, plant m^-2^), *Leymus chinensis* importance value (IV), aboveground biomass (AGB, g m^-2^), and belowground (to 10 cm depth) biomass (BGB, g m^-2^) under different treatments in July across the 3 years.

		pH	VD	IV	AGB	BGB
2012	CK	- -	461.8 ± 51.4^a^	56.7 ± 4.4^a^	217.9 ± 11.0^a^	432 ± 101^a^
	W	- -	530.2 ± 19.9^b^	72.3 ± 1.6^b^	247.8 ± 21.3^a^	305 ± 74^a^
	N	- -	718.1 ± 85.7^c^	77.7 ± 6.1^c^	371.7 ± 34.4^b^	779 ± 63^b^
	WN	- -	821.5 ± 28.2^d^	82.2 ± 1.2^c^	391.9 ± 54.9^b^	829 ± 96^b^
2013	CK	8.8 ± 0.1^a^	453.0 ± 26.4^a^	68.2 ± 4.5^a^	252.1 ± 24.4^a^	643 ± 85^a^
	W	8.6 ± 0.2^a^	565.5 ± 23.2^b^	73.4 ± 2.1^b^	251.6 ± 32.7^a^	532 ± 106^a^
	N	8.6 ± 0.2^a^	726.2 ± 48.0^c^	76.3 ± 2.4^b^	529.7 ± 28.6^b^	843 ± 55^b^
	WN	8.7 ± 0.1^a^	710.5 ± 22.5^c^	78.0 ± 5.4^b^	480.1 ± 67.9^b^	1092 ± 115^c^
2014	CK	8.9 ± 0.6^a^	445.4 ± 20.8^a^	66.7 ± 1.5^a^	202.7 ± 20.6^a^	861 ± 166^b^
	W	9.1 ± 0.4^a^	487.1 ± 45.5^a^	71.8 ± 4.4^ab^	270.6 ± 36.5^b^	468 ± 64^a^
	N	9.0 ± 0.5^a^	851.5 ± 25.3^b^	76.5 ± 2.8^b^	457.2 ± 19.0^c^	970 ± 123^bc^
	WN	9.2 ± 0.1^a^	988.8 ± 44.1^c^	77.0 ± 6.7^b^	499.4 ± 52.1^c^	1135 ± 279^c^

Values are presented as mean ± 1 SD (*n* = 6). Different letters in a column indicate significant difference among treatments at the *P* < 0.05 level (Duncan test). CK: control; W: water addition; N: nitrogen addition; WN: water and nitrogen added.

### Vegetation and biomass

Plant community density in the N and WN plots was significantly higher than in the control plots (Table 1). The effects of water addition on plant community density were significant in 2012 and 2013, but not in 2014 (Table 1). Both water and nitrogen addition increased the importance value of *Leymus chinensis*; however, the magnitude of enhancement was greater in the N plots than in the W plots (Table 1).

Aboveground biomass (AGB) and belowground biomass (BGB) were significantly higher in the N and WN plots than in the control plots (Table 1). The stimulating effects of water addition were significant only in 2014. No interactive effects were detected between water and nitrogen addition on either AGB or BGB (Tables 1 & 2).

**Table 2:** Results (*F* and *P* values) of a three-way ANOVA on the effects of year (Y), nitrogen addition (N), water addition (W), and their interactions on aboveground biomass (AGB, g m^-2^), net ecosystem CO_2_ exchange (NEE, μmol m^-2^ s^-1^), ecosystem respiration (ER, μmol m^-2^ s^-1^), and gross ecosystem productivity (GEP, μmol m^-2^ s^-1^).

		AGB		NEE		ER		GEP	
	*df*	*F*	*P*	*F*	*P*	*F*	*P*	*F*	*P*
Y	2	1.11	0.42	5.55	0.18	10.05	0.11	10.18	0.13
N	1	42.37	**0.02**	33.90	**0.03**	71.88	**0.01**	55.45	**0.02**
W	1	0.62	0.52	8.01	0.11	0.45	0.57	1.37	0.36
Y × N	2	33.00	**0.03**	9.69	0.09	1.24	0.45	2.03	0.33
Y × W	2	16.58	0.06	0.26	0.80	2.77	0.27	1.29	0.44
N × W	1	6.02	0.13	0.44	0.57	0.06	0.83	0.26	0.66
Y × N × W	2	0.48	0.62	2.83	0.07	10.82	**<0.01**	11.51	**<0.01**

The bold numerals highlight significance at the *P* < 0.05 level.

### Seasonal dynamics of ecosystem C fluxes

**Fig 3:**
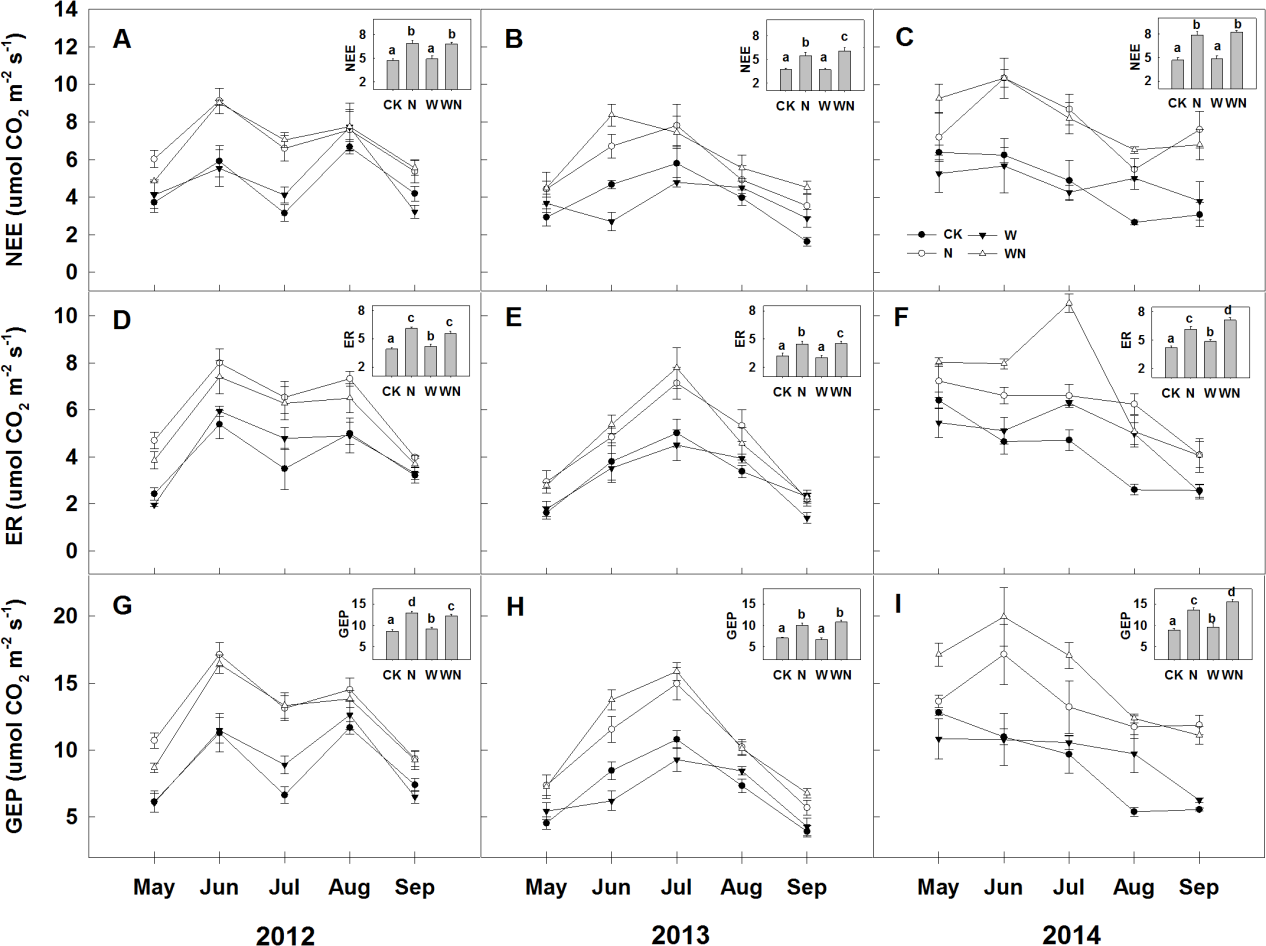
Seasonal dynamics and means of (A, B, and C) net ecosystem CO_2_ exchange (NEE, μmol m^-2^ s^-1^), (D, E, and F) ecosystem respiration (ER, μmol m^-2^ s^-1^), and (G, H, and I) gross ecosystem productivity (GEP, μmol m^-2^ s^-1^) in 2012, 2013, and 2014. Different lowercase letters indicate significant differences (*P* < 0.05) in seasonal averages among treatments (Duncan’s test). CK: control; W: water addition; N: nitrogen addition; WN: water and nitrogen added in combination. Data are reported as mean ± 1 SD (*n* = 6).

For the 3 experimental years, ecosystem C fluxes showed apparent seasonal patterns, with values of NEE, GEP, and ER being greater in the middle and lower in the beginning and end of the growing season (Fig 3). Despite the large interannual variation in growing season precipitation, there were no significant differences in ecosystem C fluxes among the 3 experimental years (Table 2).

### Effects of water and nitrogen addition on ecosystem C fluxes

Water addition enhanced ER and GEP in 2012 (by 7.3 and 5.7%, respectively) and 2014 (by 16.3 and 8.5%, respectively), but not in 2013 (Fig 3). Contrary to our expectations, water addition had no effects on NEE (Fig 3). Once we pooled the 3 years’ data together, there were no statistically significant effects of water addition on ecosystem C fluxes (Table 2).

Compared to the CK plots, nitrogen addition enhanced NEE (46.7%, 45.8%, and 68.9%), ER (56.5%, 39.2%, and 47.0%), and GEP (49.6%, 41.9%, and 52.1%) in 2012, 2013, and 2014, respectively (Fig 3). Interactive effects between nitrogen and water addition were significant in 2012 on GEP, in 2013 on NEE and ER, and in 2014 on ER and GEP. However, after we pooled the 3 years’ data together, there were no significant interactive effects between nitrogen and water addition on ecosystem C fluxes (Table 2).

### Dependence of ecosystem C fluxes on AGB or precipitation

**Fig 4:**
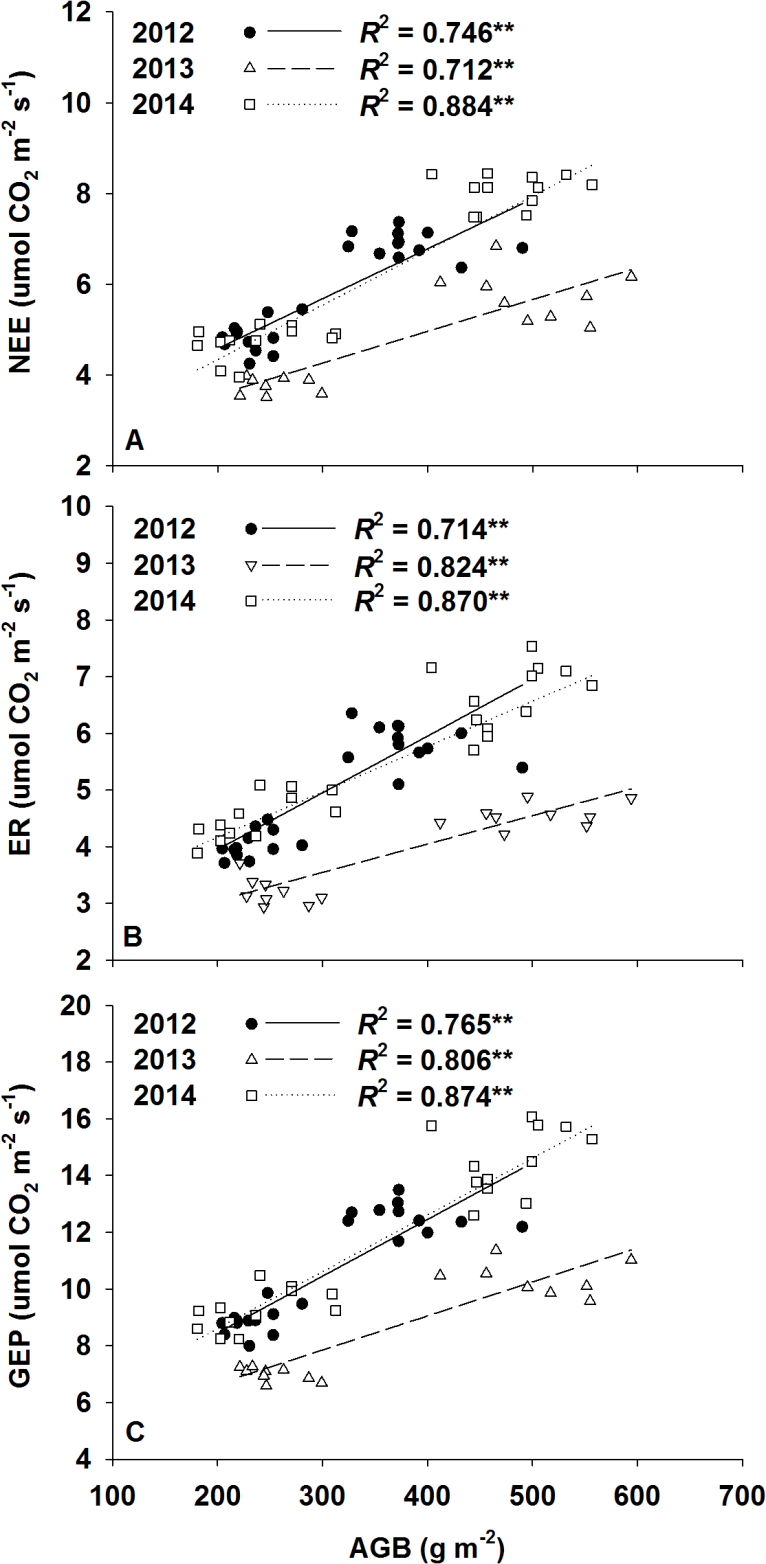
Dependence of growing season mean (A) net ecosystem CO_2_ exchange (NEE, μmol m^-2^ s^-1^), (B) ecosystem respiration (ER, μmol m^-2^ s^-1^), and (C) gross ecosystem productivity (GEP, μmol m^-2^ s^-1^) on aboveground biomass (AGB) across treatments in 2012 (solid circles), 2013 (open triangle), and 2014 (open square). ** represents a significant relationship at the *P* < 0.01 level.

**Fig 5:**
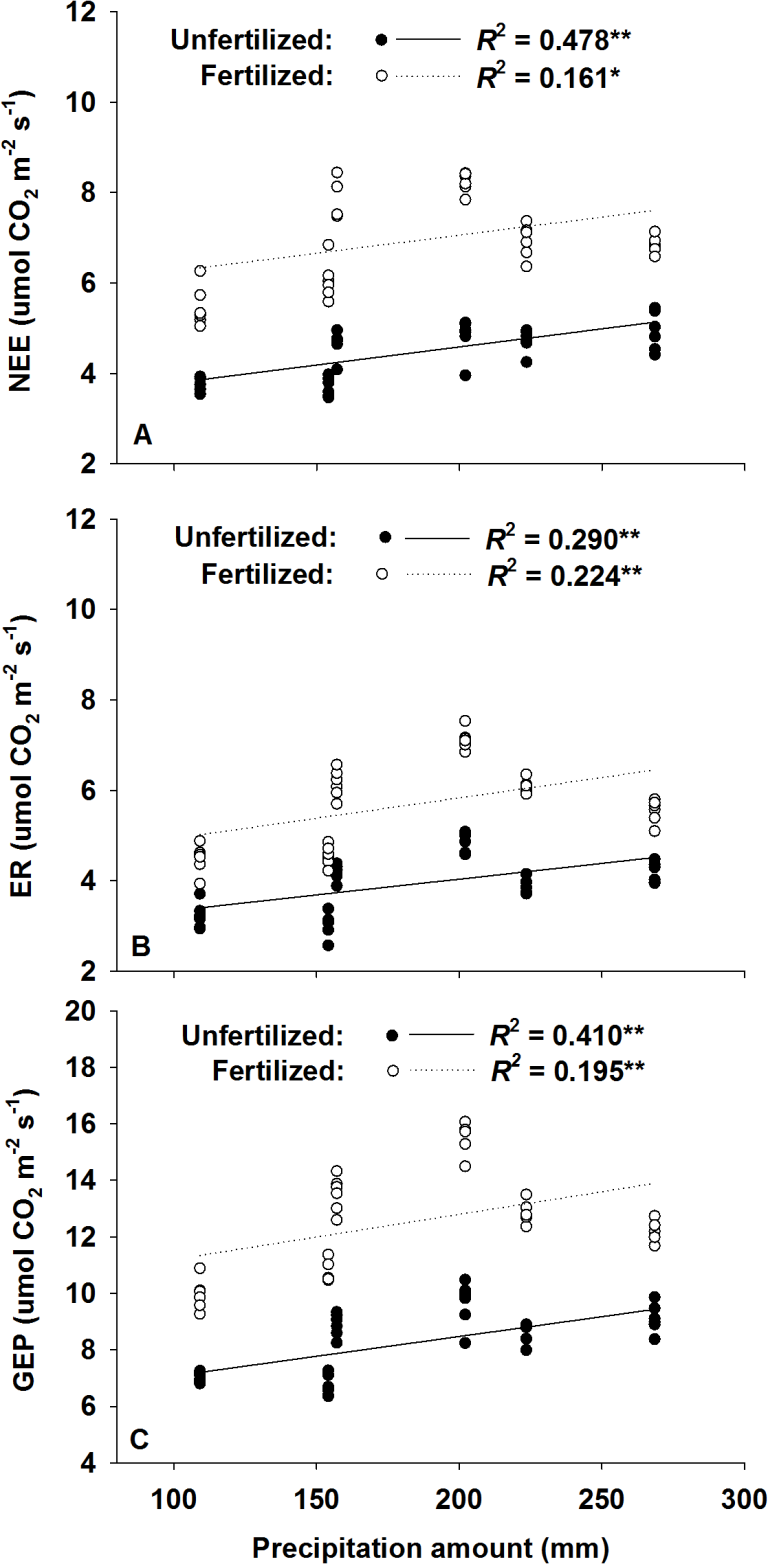
Positive dependence of growing season mean (A) net ecosystem CO_2_ exchange (NEE, μmol m^-2^ s^-1^), (B) ecosystem respiration (ER, μmol m^-2^ s^-1^) and (C) gross ecosystem productivity (GEP, μmol m^-2^ s^-1^) on the amount of early growing season (Apr, May, Jun) precipitation (water added + natural precipitation) across the 3 years in both fertilized (open circles) and unfertilized plots (solid circles). Nitrogen fertilized plots include both the N and WN plots, and unfertilized plots include both the CK and W plots. Water addition was treated as a precipitation event and included in the calculation of the precipitation amount. * and ** represent significant relationships at the *P* < 0.05 and *P* < 0.01 levels, respectively.

**Fig 6:**
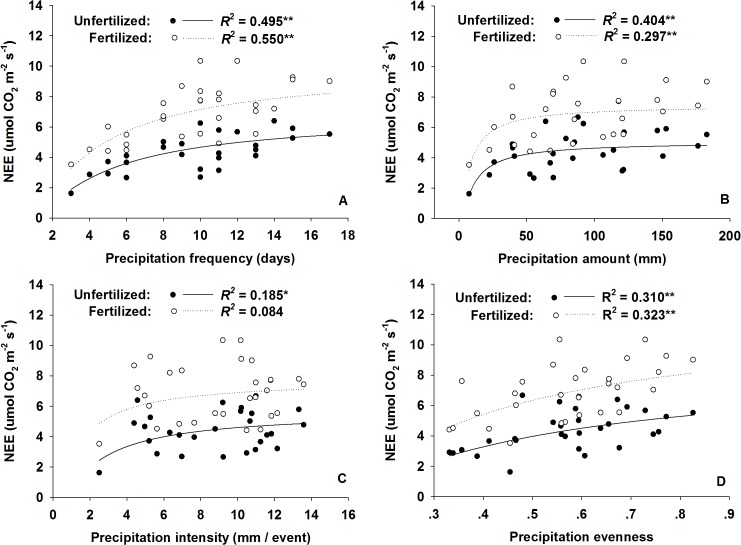
Responses of net ecosystem CO_2_ exchange (NEE, μmol m^-2^ s^-1^) to monthly precipitation regimes (A, frequency; B, amount; C, intensity; D, evenness) across the 3 years for both nitrogen fertilized (open circles, A: y = Exp(2.240 – 2.662x^-1^); B: y = Exp(2.017 – 6.269x^-1^); C: y = Exp(2.053 – 1.190x^-1^); D: y = Exp(2.479 – 0.316x^-1^)) and unfertilized plots (solid circles, A: y = Exp(1.905 – 3.610x^-1^); B: y = Exp(1.613 – 9.101x^-1^); C: y = Exp(1.700 – 1.983x^-1^); D: y = Exp(2.141 – 0.381x^-1^)). Nitrogen fertilized plots include both N and WN plots, and unfertilized plots include both CK and W plots. Water addition was treated as a precipitation event and included in the calculation of monthly precipitation regimes. * and ** represent significant relationships at the *P* < 0.05 and *P* < 0.01 levels, respectively.

Across the 3 experimental years, growing season ecosystem C fluxes were positively correlated with AGB (Fig 4). There were no significant correlations between NEE and the amount of annual precipitation; however, we did observe a positive dependence of seasonal ecosystem C fluxes on the amount of early growing season (Apr, May, and Jun) precipitation in both the nitrogen-fertilized and unfertilized plots (Fig 5). Significant nonlinear regressions (Sigmoid Curve: y = Exp (ax^-1^ + b)) between monthly NEE and monthly precipitation regimes (frequency, amount, intensity, and evenness) were also detected (Fig 6).

## Discussion

### Effects of precipitation regimes on ecosystem C fluxes

In drought-prone regions, water availability is usually the most important environmental factor controlling ecosystem CO_2_ exchange [11–13, 42]. Contrary to our expectation, we found that water addition had no impact on NEE in the studied meadow steppe. Similar results have also been reported in more humid grassland ecosystems [12, 15, 43]. These results suggest that precipitation addition does not necessarily lead to increased C uptake or release in terrestrial ecosystems [11]. The observed minimal effects of water addition on ecosystem CO_2_ exchange may be because natural precipitation in these ecosystems meets the requirements of the local plant community.

In addition to the precipitation amount, the seasonal distribution of precipitation also plays an important role in regulating NEE and C sequestration [16, 24]. In our study, there were significant relationships between annual ecosystem CO_2_ fluxes (NEE, ER, and GEP) and the early growing season (Apr, May, and Jun) precipitation input (water added + natural rainfall) (Fig 5), which could be attributed to the seasonal water demand of C_3_ species in the Songnen meadow steppe [16]. The temporal distribution of C_3_ species’ growth cycles has been observed in many previous studies [44, 45], which have found that C_3_ species flourish in the cool spring and autumn [16]. This phenomenon can be partly explained by C_3_ species having lower temperature optima for photosynthesis [46]. In the Songnen meadow steppe, vegetation is dominated by *Leymus chinensis*, which represents approximately 85% of the total plant biomass [32, 47]. Therefore, ecosystem CO_2_ exchange in the Songnen meadow steppe was highly influenced by the growth status of the *L*. *chinensis* community. As a C_3_ perennial rhizomatous grass, *L*. *chinensis* generally turns green earlier and matures before the hot summer, which makes it highly tolerant to mid- to late-growing season drought. Indeed, we found that annual ecosystem CO_2_ fluxes were affected more by the early growing season (Apr, May, and Jun) than the total growing season (Apr–Oct) precipitation (Fig 5). This highlights the importance of seasonal precipitation distribution, especially in ecosystems where the dominant species has specific seasonal water requirements.

There is now abundant experimental evidence that alterations in precipitation intensity and frequency, in addition to amount, can affect C cycling processes in terrestrial ecosystems [11, 43, 48, 49]. After incorporating water addition into the calculation of precipitation regimes (amount, frequency, and intensity), we observed that NEE correlated with both the precipitation amount and frequency for both the fertilized and unfertilized plots (Fig 6). For the studied meadow steppe, precipitation frequency plays a more important role in controlling NEE than either precipitation amount or intensity. Higher precipitation frequency is often associated with greater evenness in rainfall distribution, which reduces the fluctuation in soil moisture and promotes plant growth. In a precipitation manipulation experiment, Harper et al. (2005) and Knapp et al. (2008) found that longer precipitation intervals and greater intensity increased soil moisture fluctuations and the duration and severity of soil water stress in a mesic ecosystem [24, 48]. As a result of the lower soil permeability in the Songnen meadow steppe, a large fraction of the heavy rainfall may be lost as surface runoff; therefore, plant growth benefits more from homogenized precipitation than uneven precipitation distribution.

### Effects of nitrogen addition on ecosystem C fluxes

With a history of low atmospheric nitrogen inputs, grassland carbon dynamics are likely very sensitive to N enrichment [50–52]. However, previous studies have had contradictory results [7, 34, 35, 53]. These inconsistent findings may have resulted from differences in experimental duration or the amount and frequency of nitrogen addition. For example, Zhang et al. (2014) recently reported that observed nitrogen deposition effects may be exaggerated if nitrogen addition is applied at low frequency [52]. In our study, 5 g N m^-2^ in the form of urea was applied twice a year, which is a saturating rate for grassland ecosystems [54]. This nitrogen addition frequency is not enough to simulate natural nitrogen deposition; however urea is a slow-release organic nitrogen fertilizer, which partially makes up for such a defect.

In contrast to the lack of change in NEE under precipitation addition, increased nitrogen notably enhanced NEE, which was largely due to greater N-induced increases in GEP than in ER (Figs 2 & 3). This is in line with previous studies conducted in a tall-grass prairie [51] and a temperate steppe [34]. We observed a strong correlation between the growing season mean NEE and AGB across the 3 experimental years (Fig 4), which suggests that the stimulation of AGB may underlie the observed positive response of NEE to N addition [55]. Enhanced AGB resulted in more photosynthetic units and higher LAI, which can no doubt stimulate GEP [32]. ER may also be promoted with enhanced AGB and BGB, but a reduced root/shoot ratio weakens the stimulation of ER by nitrogen addition. Moreover, the temporal match between N supply and photosynthetic N requirements cannot be ignored. The results of Niu et al. (2005, 2008) suggest that the temporal synchronicity of water and N resources with photosynthesis is critical for the growth and biomass production of C_3_ species [16, 22]. In our study, half of the nitrogen addition was implemented during the main vegetative growth period of the *L*. *chinensis* community, which increased biomass and ecosystem carbon exchange.

The anticipated interactive effects of water and nitrogen addition on NEE were not observed in the studied humid meadow steppe (Table 2). Similar results have also been reported in a more drought-prone steppe [9], where either water addition or nitrogen addition can stimulate plant growth, but no additive effects were detected. The studied meadow steppe is located on the eastern edge of the Eurasian temperate steppe, where mean annual precipitation is slightly higher than the temperate steppe in Inner Mongolia. The effects of water addition treatment may be diminished by the higher background precipitation. Furthermore, the stimulation of nitrogen addition on NEE in the studied meadow steppe may be constrained by high soil pH and salinization [47], which often reduce the activities of mycorrhizal fungi and rhizosphere bacteria [56–58]. Water addition had no impacts on soil pH or salinization (Table 1); therefore, it did not relieve the potential constraint of soil pH on nitrogen availability.

## Conclusions

Ecosystem C fluxes in the Songnen meadow steppe in northeast China were significantly affected by nitrogen addition, but not by water addition. The consistent positive nitrogen effects on NEE from 2012 to 2014 (which differed greatly in precipitation regimes) suggest that nitrogen deposition steadily increases C sequestration in a less water-limited meadow steppe. The responses of NEE to precipitation regimes were dominated by frequency rather than amount or intensity. These findings highlight the critical roles that precipitation distribution and associated soil moisture fluctuations play in regulating ecosystem C fluxes in humid grasslands. Overall, future ecosystem productivity in the studied meadow steppe will benefit more from the predicted increase in N deposition than from greater total precipitation.
